# The Effects of Endometrial Thickness on Pregnancy Outcomes of Fresh IVF/ICSI Embryo Transfer Cycles: An Analysis of Over 40,000 Cycles Among Five Reproductive Centers in China

**DOI:** 10.3389/fendo.2021.788706

**Published:** 2022-01-24

**Authors:** Jianing Xu, Shaodi Zhang, Lei Jin, Yundong Mao, Juanzi Shi, Rui Huang, Xiao Han, Xiaoyan Liang, Cuilian Zhang

**Affiliations:** ^1^ Reproductive Medicine Center, Henan Provincial People’s Hospital, People’s Hospital of Zhengzhou University, Zhengzhou, China; ^2^ Reproductive Medicine Center, Tongji Hospital of Tongji Medical College of Huazhong University of Science and Technology, Wuhan, China; ^3^ Reproductive Medicine Center, Jiangsu Provincial Hospital, Nanjing, China; ^4^ Reproductive Medicine Center, Northwest Women’s and Children’s Hospital, Xi’an, China; ^5^ Reproductive Medicine Center, Sixth Affiliated Hospital of Sun Yat-sen University, Guangzhou, China

**Keywords:** IVF/ICSI, clinical pregnancy rate, endometrial thickness, live birth rate (LBR), fresh embryo transfer (fresh ET)

## Abstract

**Objective:**

To investigate the effects of endometrial thickness (EMT) on pregnancy outcomes on hCG trigger day in fresh *in vitro* fertilization (IVF) and intracytoplasmic sperm injection (ICSI) cycles.

**Methods:**

A total of 42,132 fresh cycles between 1 January 2013 and 31 December 2019 were included in this retrospective cohort study. Data were collected from five reproductive centers of large academic or university hospitals in China. All patients were divided into different groups according to their endometrial thickness on hCG trigger day. Multivariate regression analysis, curve fitting and threshold effect analysis were performed.

**Results:**

After adjusting for age, body mass index, infertility type, number of embryos transferred, number of retrieved oocytes and COS (controlled ovarian stimulation) protocols, significant associations were found between endometrial thickness and clinical pregnancy rate (adjusted odds ratio [aOR]: 1.05; 95% confidence interval [CI]: 1.06–1.08, P < 0.0001), live birth rate (aOR: 1.04; 95% CI: 1.03–1.05, P < 0.0001) as well as miscarriage rate(aOR: 0.96; 95% CI: 0.94 – 0.98, P < 0.0001). When the endometrial thickness was less than 12mm, the clinical pregnancy rate and live birth rate were increased significantly by 10% and 9%(OR:1.10; 95%CI: 1.08-1.12, OR:1.09; 95%CI: 1.07-1.11), respectively, along with the increase of each millimeter increment of endometrial thickness. However, when the EMT ranged from 12-15 mm, were stable at the ideal level, that were not significantly associated with EMT growth. Additionally, clinical pregnancy rate and live birth rate were slightly reduced by 6% and 4% when EMT was ≥15mm. Meanwhile, the miscarriage rate was significantly declined by 8% (OR:0.92; 95%CI: 0.90-0.95)with each millimeter increment of EMT. And when EMT was thicker than 12mm, the miscarriage rate didn’t change any more significantly.

**Conclusions:**

Endometrial thickness exhibits a curvilinear relationship with pregnancy outcomes in fresh embryo transfer cycles. Clinical pregnancy rate, live birth rate and miscarriage rate may achieve their optimal level when EMT ≥ 12 mm, but some adverse pregnancy outcomes would be observed when EMT ≥15 mm especially for clinical pregnancy.

## Introduction

Steroid hormones secreted from the ovaries regulate cell division, differentiation and degeneration in human endometrium. A woman’s menstrual cycle is divided into menstrual phase, proliferation phase, and secretory phase (1). In the proliferation phases, the endometrium increases in preparation for embryo implantation (2). In the secretory phase, the functional layer of the endometrium thickens in preparation for embryo implantation (3).

Endometrial receptivity is widely accepted as one of the key factors affecting ART(assisted reproductive technology) outcomes. Generally, ultrasound examination is carried out as routine method of endometrium evaluation in IVF cycles in most reproductive medicine agencies due to its convenient and noninvasive property (4). Sonographic parameters, such as endometrial thickness (EMT), endometrial pattern, endometrial volume and endometrial or sub-endometrial blood flow are used to identify endometrial receptivity (5).

Previous studies have demonstrated that increased EMT on the day of embryo transfer (ET) resulted in better pregnancy outcomes after fresh cycles (6, 7). However, other studies showed that EMT on the day of ET is a poor predictor of IVF outcomes (8, 9). Although there have been numerous studies investigating the relationship between EMT and pregnancy outcomes, there is still no consensus. The inconsistency of the results may attribute to many potential confounders such as maternal age, stimulation protocol used, numbers of egg retrieved and embryos transferred.

Therefore, in order to investigate the relationship between EMT and pregnancy outcomes including clinical pregnancy rate, live birth rate, and miscarriage rate, we designed this study which included 42,132 cycles collected from five IVF centers in China between 1 January 2013 and 31 December 2019.

## Materials and Methods

### Study Cohort

**Figure 1:**
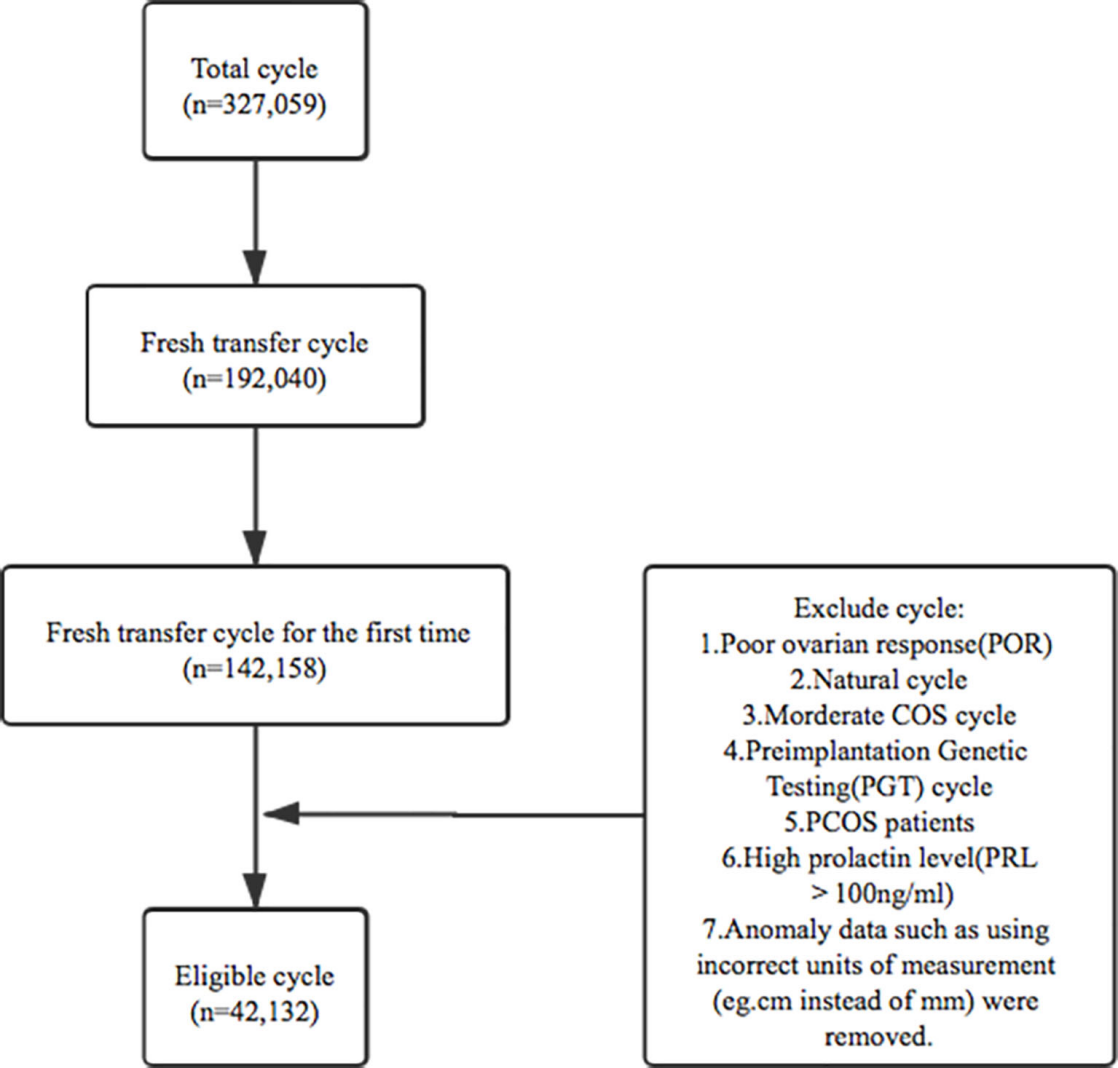
The flowchart for the screening process.

This study was a retrospective cohort analysis of five reproductive centers in university-affiliated hospitals or large academic hospitals in China including the Sixth Affiliated Hospital of Sun Yat-sen University, Henan Provincial People’s Hospital, Jiangsu Provincial People’s Hospital, Tongji Hospital of Tongji Medical College of Huazhong University of Science and Technology and Northwest Women’s and Children’s Hospital. The inclusion criteria were: 1. patients undertaken *in vitro* fertilization (IVF) and intracytoplasmic sperm injection (ICSI) between 1 January 2013 and 31 December 2019; 2. controlled ovarian stimulation (COS) cycles; 3. fresh transfer cycle for the first time; 4. endometrium was thicker than 5mm on hCG trigger day. Patients were excluded if they met any of these following criteria:1.poor ovarian response(POR) cycle; 2.natural cycle; 3.moderate COS cycle; 4. preimplantation genetic testing(PGT) cycle; 5. PCOS patients; 6.high prolactin level(PRL>100ng/ml); 7. some clinics reported that their input data using incorrect units of measurement (cm instead of mm), since it was not clear how many cycles had been affected, data from these clinics were removed. After the corresponding standard screening (listed in **Figure 1**), a number of 42,132 eligible fresh cycles between 1 January 2013 and 31 December 2019 were included.

The study was reviewed and approved separately by the ethical committees in each hospital, including Reproductive Medicine Ethics Committee of Henan Provincial People’s Hospital(SYSZ-LL-2019110401), Medical Ethics Committee of Tongji Hospital of Tongji Medical College of Huazhong University of Science and Technology(TJ-IRB20210320), Medicine Ethics Committee of Jiangsu Provincial People’s Hospital (2020-SR-046), Medical Ethics Committee of the Sixth Affiliated hospital of Sun Yat-sen University (2020ZSLYEC-295), and Medical Ethics Committee of Northwest Women’s and Children’s Hospital (2019013), and the Ethics Committee of People’s Hospital of Zhengzhou University.

### Patient Data Collection

For fresh IVF/ICSI embryo transfer cycles, the EMT on the day of hCG trigger was recorded. Analysis was restricted to fresh embryo transfer cycles after ovarian stimulation in accordance with clinical practice rules at the five centers.

All patients were grouped according to the EMT on hCG trigger day. There were nine groups for EMT between 5mm to 15mm with a 1 mm interval between each group, while all the cycles with EMT≥15mm were grouped together. In total, this study held eleven different patient groups with varying levels of pregnancy outcomes.

There were three main outcomes of interest, clinical pregnancy rate, live birth rate and miscarriage rate. Clinical pregnancy was defined as the presence of a gestational sac on first trimester ultrasound. Live birth was defined as the delivery of a live baby after 24 weeks of gestation. Miscarriage was defined as clinical intrauterine pregnancy loss before 12 weeks of gestation.

Prior to embryo transfer, each embryo was graded based on developmental speed, degree of fragmentation and the evenness of cleavage sphere. The embryos with 7-9 blastomere, uniform cytoplasm, regular morphology, fragmentation < 10% were considered to be high-quality embryos. Blastocyst scoring was performed following the Gardner scoring system [8], and embryos graded 3BB or higher were defined as good quality embryos.

### Outcome and Statistical Analysis

Clinical pregnancy rate, live birth rate and miscarriage rate were calculated per fresh ET cycle. The calculation formula used were: clinical pregnancy rate = number of cycles with clinical pregnancy/number of fresh ET cycles* 100%; live birth rate = number of cycles with live births/number of fresh ET cycles*100%; miscarriage rate = number of cycles with miscarriage/number of cycles with clinical pregnancy* 100%.

Statistical analysis was performed using the Empower Stats software base on R language. Continuous variables were presented as mean ± SD or median (Q1-Q3), and categorical variables were presented as N (%). Comparisons of these variables between groups were performed using the one-way ANOVA and χ2 tests for categorical variables. Multivariate regression analysis, curve fitting and threshold effect analysis were performed on all cycles. Confounders were selected on the basis of their association with the outcomes of interest or a change in effect estimate of 10%. Smooth curve fitting was performed to assess if there was any non-linear relationship between EMT and pregnancy outcomes. A piece wise linear regression method was used to analyze the threshold effect between EMT and pregnancy outcomes. The statistical significance was defined as a P-value less than 0.05 in the results.

## Results

### Baseline and Patient’s Characteristics

A total of 42,132 IVF/ICSI fresh embryo transfer cycles were included in our study. The baseline characteristics included female age, BMI, infertility type, basal AMH, basal FSH, basal E2, duration of infertility, AFC, COS protocols, Gn dosage, Gn duration, E2 level, LH level and P level on hCG trigger day, EMT, No. of oocytes retrieved, No. of embryos transferred, embryo type and embryo quality. The overall clinical pregnancy rate per embryo transfer cycle was 57.08% (24,048/42,132), the overall live birth rate per embryo transfer cycle was 43.30%(18,243/42,132), and the spontaneous abortion rate per pregnancy was 12.64% (3,039/24,048). Patient demographics and characteristics are shown in **Table 1**.

**Table 1:** Characteristics of patients undergoing fresh cycles with embryo transfer.

	Mean (SD) or Median (Q1-Q3)	N (%)
Female age(year)	31.16 (4.39)	
Female age group(year)		
<35		32,816 (77.89%)
>=35, <40		7,694 (18.26%)
>=40		1,622 (3.85%)
BMI (kg/m2)	22.34 (3.12)	
BMI group(kg/m2)		
<18.5		3,315 (7.91%)
>=18.5, <24.9		30,712 (73.28%)
>=24.9, <30		7,064 (16.86%)
>=30		819 (1.95%)
Infertility type		
Primary Infertility		22,505 (54.37%)
Secondary Infertility		18,887 (45.63%)
Basal AMH(ng/ml)	3.13 (1.92-4.98)	
Basal FSH(mIU/ml)	7.30 (1.99)	
Basal E2(pg/ml)	45.88 (25.79)	
Duration of infertility(year)	3.00 (2.00-5.00)	
AFC	11.71 (4.92)	
COS protocols		
GnRH agonist protocol		34,388 (81.62%)
GnRH antagonist protocol		7,744 (18.38%)
Gn dosage(IU)	2389.14 (892.99)	
Gn duration(day)	10.36 (2.18)	
E2 level on hCG trigger day(pg/ml)	2263.00 (1348.89-3571.75)	
LH level on hCG trigger day(mIU/ml)	1.73 (1.05-2.70)	
P level on hCG trigger day(ng/ml)	0.94 (0.65-1.35)	
EMT on hCG trigger day (mm)	11.61 (2.45)	
No. of retrieved oocytes	9.75 (4.53)	
No. of embryo transfer	1.61(0.49)	
Embryo type		
Embryos in cleavage stage		33,476 (79.48%)
Blastocyst		8,459 (20.08%)
others		182 (0.43%)
Embryo quality		
good		30,039 (71.62%)
Not good		11,886 (28.34%)
others		19 (0.05%)
Miscarriage rate(%)		3039 (12.64%)
Live birth rate(%)		18,243 (43.30%)
Clinical pregnancy rate(%)		24,048 (57.08%)

BMI, body mass index; AMH, anti-Mullerian hormone; AFC, antral follicle counting; controlled COS, ovarian stimulation; GnR,H gonadotropin-releasing hormone; Gn, gonadotropin; E2, estradiol; LH, luteinizing hormone; P, progesterone; EMT, endometrium thickness.

### Comparison of Clinical Outcomes Between Different Groups

Patients were divided into eleven different groups according to their EMT on hCG trigger day. The clinical pregnancy rate and live birth rate were significantly different among groups with different EMT (P < 0.001). It was noteworthy that with the increased EMT, the percentage of clinical pregnancy and live birth also increased. Whereas, there was no significant difference in the miscarriage rate among groups (P > 0.05). The information was detailed in **Table 2**.

**Table 2:** Pregnancy outcomes by EMT.

EMT(mm)	5-5.9	6-6.9	7-7.9	8-8.9	9-9.9	10-10.9	11-11.9	12-12.9	14-14.9	15-15.9	>=15	P
N	112	476	1,281	2,985	4,582	6,370	6,434	6,411	4,784	3,216	4,014	
Clinical pregnancy	38 (33.93%)	191 (40.13%)	561 (43.79%)	1,471 (49.28%)	2,500 (54.56%)	3,511 (55.12%)	3,749 (58.27%)	3,868 (60.33%)	2,904 (60.70%)	1,953 (60.73%)	2,498 (62.23%)	<0.001
Live birth	24 (21.43%)	129 (27.10%)	399 (31.15%)	1,096 (36.72%)	1,844 (40.24%)	2,634 (41.35%)	2,861 (44.47%)	2,955 (46.09%)	2,234 (46.70%)	1,509 (46.92%)	1,928 (48.03%)	<0.001
Miscarriage	9 (23.68%)	39 (20.42%)	99 (17.65%)	227 (15.43%)	361 (14.44%)	461 (13.13%)	455 (12.14%)	465 (12.02%)	335 (11.54%)	229 (11.73%)	296 (11.85%)	<0.001

### Univariate Analysis of Factors Associated With Clinical Outcomes

Univariate analysis showed that EMT was associated with an increased clinical pregnancy rate (OR=1.07, 95% CI: 1.06-1.08, P<0.0001) and increased live birth rate (OR=1.06, 95% CI: 1.06-1.07,P<0.0001). While the miscarriage rate was not significantly associated with EMT (OR=0.99, 95% CI: 0.97-1.00, P=0.0937). Female age, duration of infertility, LH levels, and P levels showed decreased effect on clinical pregnancy, live birth. However, basal AMH, AFC, Gn duration, No. of retrieved oocytes and No. of embryos transferred could increase the rate of pregnancy and live birth. The analysis data was showed in **Table 3**.

**Table 3:** Univariate analysis of factor associated with clinical outcomes.

Item	Clinical pregnancy		Live birth		Miscarriage	
	OR,95%CI	*P*	OR,95%CI	*P*	OR,95%CI	*P*
Female age	0.94 (0.93, 0.94)	<0.0001	0.93 (0.92, 0.93)	<0.0001	1.10 (1.09, 1.11)	<0.0001
BMI	1.01 (1.00, 1.02)	0.0006	1.00 (0.99, 1.01)	0.9242	1.04 (1.03, 1.05)	<0.0001
Duration of infertility(year)	0.97 (0.96, 0.97)	<0.0001	0.97 (0.96, 0.97)	<0.0001	1.05 (1.04, 1.06)	<0.0001
Infertility type						
Primary Infertility	Ref.		Ref.		Ref.	
Secondary Infertility	0.86 (0.83, 0.90)	<0.0001	0.87 (0.83, 0.90)	<0.0001	1.35 (1.25, 1.46)	<0.0001
Basal AMH(ng/ml)	1.05 (1.04, 1.07)	<0.0001	1.08 (1.07, 1.10)	<0.0001	0.96 (0.94, 0.98)	0.0008
AFC	1.03 (1.03, 1.04)	<0.0001	1.03 (1.03, 1.03)	<0.0001	0.98 (0.97, 0.99)	<0.0001
COS protocols						
GnRH agonist protocol	Ref.		Ref.		Ref.	
GnRH antagonist protocol	0.58 (0.55, 0.61)	<0.0001	0.49 (0.47, 0.52)	<0.0001	1.15 (1.04, 1.27)	0.0084
Gn dosage(IU)	1.00 (1.00, 1.00)	<0.0001	1.00 (1.00, 1.00)	<0.0001	1.00 (1.00, 1.00)	<0.0001
Gn duration(day)	1.03 (1.02, 1.04)	<0.0001	1.03 (1.02, 1.03)	<0.0001	1.01 (0.99, 1.03)	0.1999
E2 level on trigger day(pg/ml)	1.00 (1.00, 1.00)	<0.0001	1.00 (1.00, 1.00)	<0.0001	1.00 (1.00, 1.00)	0.0268
LH level on hCG trigger day(mIU/ml)	0.96 (0.95, 0.98)	<0.0001	0.96 (0.95, 0.97)	<0.0001	1.01 (0.99, 1.03)	0.4798
P level on hCG trigger day(ng/ml)	0.96 (0.95, 0.98)	<0.0001	1.04 (1.02, 1.06)	<0.0001	0.99 (0.96, 1.03)	0.5807
EMT on hCG trigger day (mm)	1.07 (1.06, 1.08)	<0.0001	1.06 (1.06, 1.07)	<0.0001	0.95 (0.94, 0.97)	<0.0001
No. of retrieved oocytes	1.04 (1.03, 1.04)	<0.0001	1.02 (1.02, 1.03)	<0.0001	0.97 (0.97, 0.98)	<0.0001
No. of embryos transferred	1.46 (1.41, 1.52)	<0.0001	1.93(1.85, 2.01)	<0.0001	0.89 (0.83, 0.97)	0.0044
Embryo type						
Embryos in cleavage stage	Ref.		Ref.		Ref.	
Blastocyst	1.37 (1.30, 1.44)	<0.0001	1.20 (1.15, 1.26)	<0.0001	1.04 (0.94, 1.14)	0.3491
others	0.06 (0.03, 0.11)	<0.0001	0.06 (0.03, 0.13)	<0.0001	0.00 (0.00, inf.)	0.9061
Embryo quality						
good	Ref.		Ref.		Ref.	
Not good	0.58 (0.56, 0.61)	<0.0001	0.56 (0.54, 0.59)	<0.0001	1.11 (1.01, 1.21)	0.0234
others	0.37 (0.15, 0.94)	0.0374	0.30 (0.10, 0.89)	0.0306	0.00 (0.00, inf.)	0.9311

BMI, body mass index; AMH, anti-Mullerian hormone; AFC, antral follicle counting; controlled COS, ovarian stimulation; GnRH, gonadotropin-releasing hormone; Gn, gonadotropin; E2, estradiol; LH, luteinizing hormone; P, progesterone; EMT, endometrium thickness.

### Associations Between EMT and Clinical Outcomes of Fresh Cycles Using Multivariable Logistic Regression Analysis

After adjusting for confounding variables such as age, BMI, infertility type, No. of embryos transferred, No. of retrieved oocytes and COS protocols. we performed a multivariable logistic regression analysis to evaluate the association between EMT and pregnancy outcomes. Significant associations were found between EMT and clinical pregnancy rate (adjusted odds ratio [aOR]: 1.05; 95% confidence interval [CI]: 1.06–1.08, P< 0.0001), live birth rate (aOR: 1.04; 95% CI: 1.03–1.05, P < 0.0001), and miscarriage rate (aOR: 0.96; 95% CI: 0.94 – 0.98, P < 0.0001).The result was exhibited in **Table 4**.

**Table 4:** Associations between EMT and pregnancy outcomes of fresh cycles using multivariable logistic regression analysis.

Outcome	Non-adjusted		Adjusted	
	OR, 95%CI	*P*	OR, 95%CI	P
Clinical pregnancy	1.07 (1.06, 1.08)	<0.0001	1.05 (1.04, 1.06)	<0.0001
Live birth	1.06 (1.06, 1.07)	<0.0001	1.04 (1.03, 1.05)	<0.0001
Miscarriage	0.95 (0.94, 0.97)	<0.0001	0.96 (0.94, 0.98)	<0.0001

Adjusted for age, BMI, infertility type, No. of embryos transferred, No. of retrieved oocytes and COS protocols.

### Curve Fitting Between EMT and Pregnancy Outcomes

**Figure 2:**
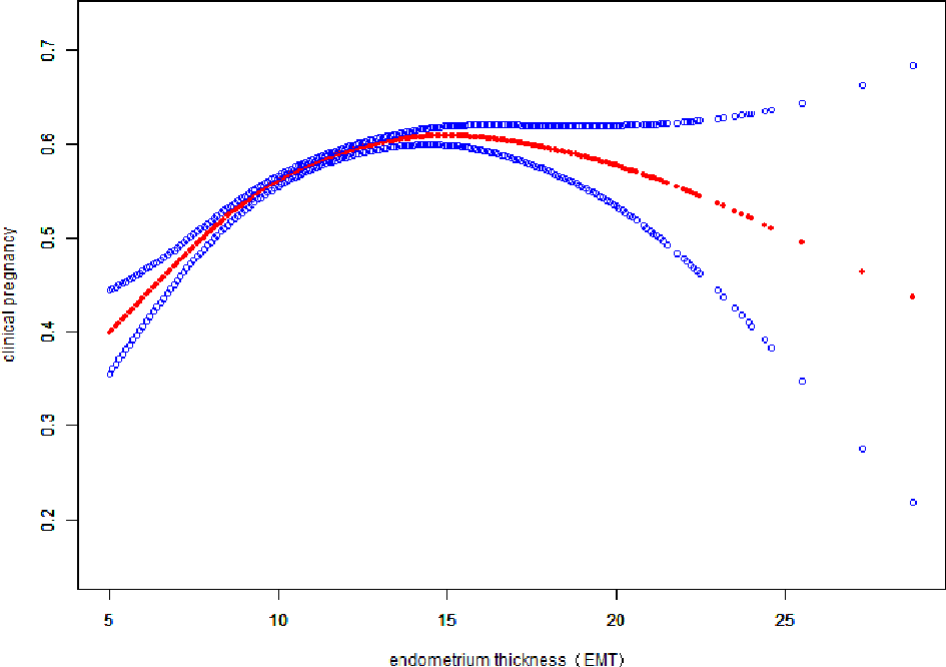
Association between EMT and clinical pregnancy rate. A threshold, nonlinear association between EMT and clinical pregnancy rate was found (P<0.001) in a generalized additive model (GAM). The solid red line represents the smooth curve fit between variables. Blue bands represent the 95% of confidence interval from the fit. The fitted line adjusted for age, BMI, infertility type, and the number type of embryos transferred, number. of retrieved oocytes, and COS protocols.

**Figure 3:**
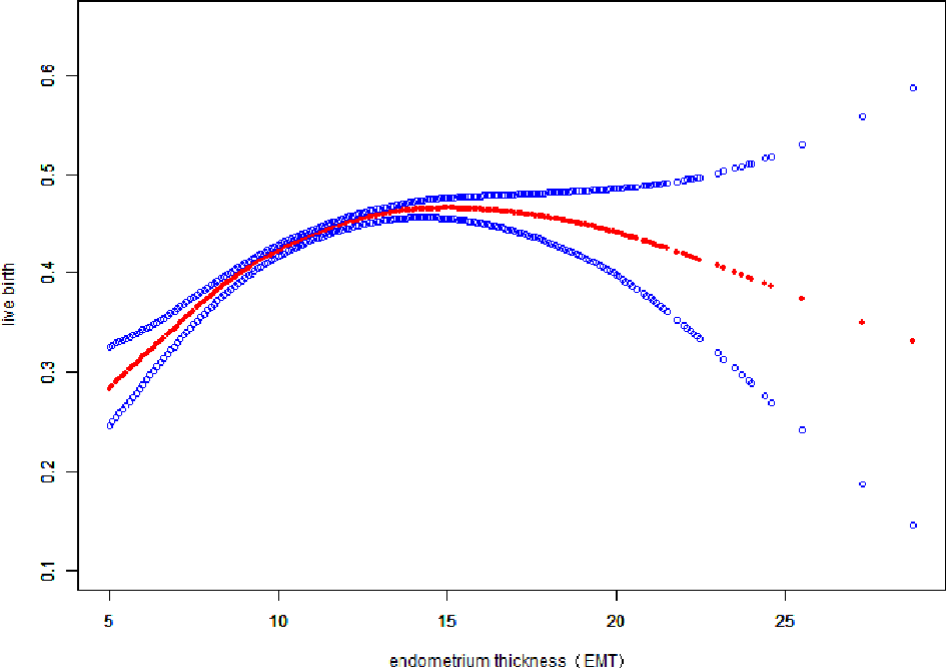
Association between EMT and live birth rate. A threshold, nonlinear association between EMT and live birth rate was found (P<0.001) in a generalized additive model (GAM). Solid red line represents the smooth curve fit between variables. Blue bands represent the 95% of confidence interval from the fit. The fitted line adjusts for age, BMI, infertility type, and the number type of embryos transferred, numbers of retrieved oocytes, and COS protocols.

**Figure 4:**
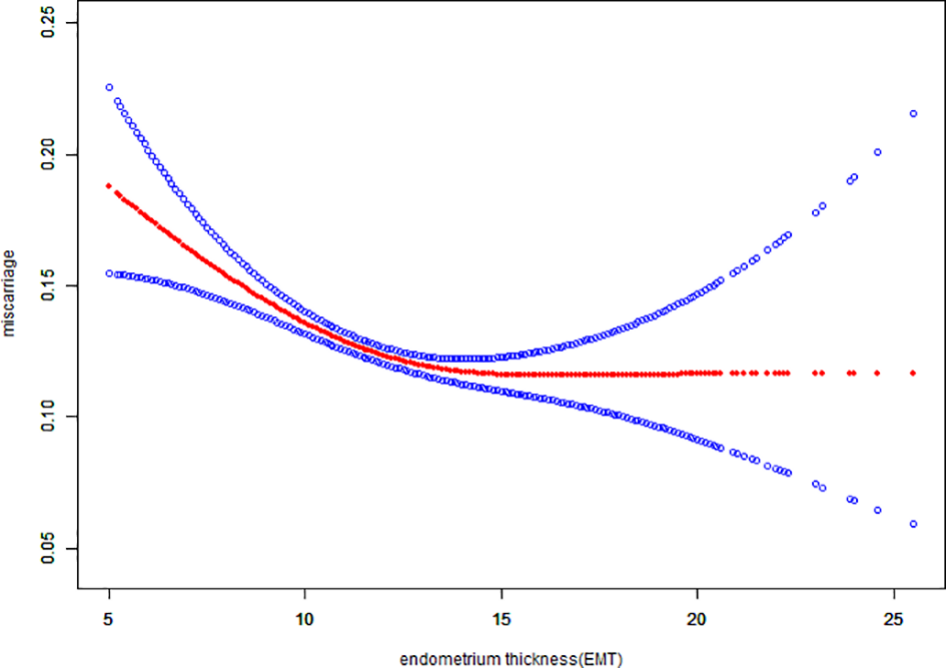
Association between EMT and miscarriage rate. A threshold, nonlinear association between EMT and miscarriage rate was found (P<0.001) in a generalized additive model (GAM). The solid red line represents the smooth curve fit between variables. Blue bands represent the 95% of confidence interval from the fit. The fitted line adjusted for age, BMI, infertility type, and the number type of embryos transferred, number. of retrieved oocytes, and COS protocols.

Fitted curves with covariate adjustment, showing the relationship between EMT and clinical pregnancy rate, live birth rate and miscarriage rate, were presented in **Figures 2**, **3**. As EMT increased, clinical pregnancy rate and live birth rate initially increased before declining at high levels (**Figures  2**, **3**), shown like an inverted “U” shape. Meanwhile, the miscarriage rate first declined and then leveled off with EMT increasing (**Figure 4**).

### Threshold Effect Analysis of EMT and Pregnancy Outcomes in Fresh Cycles Using Piece Wise Linear Regression Method

The threshold effect analysis of EMT and the pregnancy outcomes were presented in **Table 5**. The EMT was a non-linear significant predictor of pregnancy outcomes. When the EMT was less than 12mm, the clinical pregnancy rate and live birth rate were significantly increased by 10% and 9%(OR:1.10; 95%CI: 1.08-1.12, OR:1.09; 95%CI: 1.07-1.11), respectively, along with the increase of each millimeter increment of EMT. As the EMT ranged from 12 to 15 mm, the pregnancy outcomes came to the plateau phase at the ideal level. However, clinical pregnancy rate and live birth rate tended to decrease by 6% and 4% when EMT was ≥15mm. In addition, the miscarriage rate was significantly declined by 8% (OR:0.92; 95%CI: 0.90-0.95)with each millimeter increment of EMT. And when EMT was thicker than 12mm, the miscarriage rate didn’t change significantly any more.

**Table 5:** Threshold effect analysis of EMT and clinical outcomes in fresh cycles using piece wise linear regression method.

Outcomes	cutoff of EMT	OR, 95%CI	P
Clinical pregnancy	<12mm	1.10 (1.08, 1.12)	<0.0001
	12-15mm	1.00 (0.98, 1.03)	0.7863
	≥ 15mm	0.94 (0.89, 0.99)	0.0204
Live birth	<12mm	1.09 (1.07, 1.11)	<0.0001
	12-15mm	1.00 (0.97, 1.03)	0.8839
	≥ 15mm	0.96 (0.91, 1.01)	0.1094
Miscarriage	<12mm	0.92 (0.90,0.95)	<0.0001
	12-15mm	1.00 (0.94,1.05)	0.9031
	≥ 15mm	1.03 (0.93,1.15)	0.5169

## Discussion

To the best of our knowledge, this is the largest multi-center study to demonstrate the relationship between EMT and pregnancy outcomes such as clinical pregnancy rate, live birth rate and miscarriage rate. We determined cut-off values for EMT in this large cohort including 42,132 IVF/ICSI fresh embryo transfer cycles. Our study found EMT to be a non-linear significant predictor of pregnancy outcomes, including clinical pregnancy, live birth rate and miscarriage rate. With the increase of EMT, clinical pregnancy rate and live birth rate initially increased before declining at high levels. Moreover, we also found that miscarriage rates could decreased with the growth of EMT in fresh cycles.

In most reproductive medicine centers, EMT and endometrial patterns are usually considered to be reliable and noninvasive measurements in endometrial receptivity evaluation. Endometrial receptivity is the key factor affecting the pregnancy outcomes of embryo transfer cycles (10). Since the EMT has been measured at different time points during previous studies, such as on the day of hCG administration, on the day of oocyte retrieval or on the day of embryo transfer (11–13), therefore there is no consensus on the relationship between EMT and pregnancy outcomes despite the relative abundance of studies.

As far as we know, most previous studies only focused on whether EMT affects pregnancy outcomes or not. Yuan et al. (6) showed EMT as one of the independent variables predictive of clinical pregnancy (OR:1.097; P<0.001), live birth (OR:1.078; P<0.001), spontaneous abortion (OR:0.948; P<0.001), and ectopic pregnancy (OR:0.851; P<0.001). Gallos et al. (14) considered EMT is strongly associated with pregnancy losses and live births in IVF. Ma et al. (15) also investigated that live birth rate and clinical pregnancy rate were positively correlated with increasing EMT. In addition, a meta-analysis (5) including 9 prospective and 21 retrospective investigations also indicated that compared with high EMT, low EMT was associated with decreased pregnancy rate (n = 30, OR: 0.61; 95%CI: 0.52 - 0.70; P < 0.001). However, there were still some mixed conclusions. In these studies of Craciunas et al. and Huang, J et al. (8, 9), biomarkers such as EMT had poor ability to predict clinical pregnancy. In order to obtain a more comprehensive relationship between EMT and pregnancy outcomes, we adjusted for confounding factors and conducted curve fitting and threshold effect analysis.

We performed multivariable logistic regression analysis to evaluate the association between EMT and pregnancy outcomes. Significant associations were found between EMT and clinical pregnancy rate (aOR: 1.05; 95% CI: 1.06–1.08, P<0.0001), live birth rate (aOR: 1.04; 95% CI: 1.03–1.05, P<0.0001) and miscarriage rate(aOR: 0.96; 95% CI: 0.94 – 0.98, P < 0.0001), similar to results of other studies (5, 16, 17). A curve fitting and threshold analysis further revealed a quantitative relationship between EMT and pregnancy outcomes. The correlation between EMT and pregnancy outcome tended to be an inverted “U” shape. Our data suggested that clinical pregnancy rate could reach 49.28% with EMT between 8-8.9mm, which was acceptable among most IVF centers. With every millimeter increase of endometrium up to 12 mm, the clinical pregnancy rate and live birth rate increased by 10% and 9%(OR:1.10; 95%CI: 1.08-1.12, OR:1.09; 95%CI: 1.07-1.11), respectively. When EMT attained to the range of 12-15 mm, clinical pregnancy rate and live birth rate were stable at the ideal level of 60.33% - 60.73% and 46.09% - 46.92%, which were not significant associated with EMT growth. As the EMT increased thicker than 15mm, clinical pregnancy rate and live birth rate tended to be slightly decreased by 6% and 4%. So we could speculate about the minimum threshold of EMT for optimal clinical pregnancy and live birth rate may at 12 mm.

In the study of K.E. Liu et al. (18), they reported that clinical pregnancy and live birth rates decreased (P < 0.0001) and pregnancy loss rates increased (P = 0.01) with each millimeter decline in endometrial thickness below 8 mm in 24,363 fresh cycles. Zhang T et al (4) performed binary logistic analysis and ROC curves in 1,512 IVF cycles and revealed the cut-off points of endometrial thickness on oocyte retrieval day was 8.75 mm (sensitivity of 84.6% and specificity of 22.8%) for live birth. Besides, Gallos et al. (14) considered that endometrial thickness is strongly associated with pregnancy losses and live births in IVF, and the optimal endometrial thickness threshold of 10 mm. Those cut-off values in previous studies were different to ours. What should be pointed out is that the present study used smooth curve fitting, segmented regression model, and then found the non-linear relationship and the threshold value of EMT. It’s a new statistical method applied to the field of EMT and this method should be used to find the exact association when there is a possible threshold effect.

In addition, we noted that although clinical pregnancy and live birth rates were lower when the endometrial thickness was below 8mm. Clinical pregnancy and live birth rates were 33.93% to 43.79% and 21.43% to 31.15% with an endometrial thickness of 5 – 7.9mm in **Table 2**. Many studies had different definitions for thin endometrium and used different thresholds of 6-8 mm to define thin endometrium (6, 19–22).The reason for why a thin endometrium leads to implant failure or early pregnancy loss still remains unclear. For patients with a thin endometrium, endometrial receptivity may be compromised because of the alteration of implantation associated factors (23). A thinned or absent functional layer may subject the embryos to higher vascularity and oxygen concentrations from the basal endometrium (13, 23). As a result, the subsequent production of reactive oxygen species could generate a suboptimal uterine environment for implantation and placentation, which ultimately leads to impaired fetal growth (23, 24). However, a thickened endometrium (≥ 15mm, data displayed in **Table 5**) had an adverse effect on clinical pregnancy rate decreasing by 6% significantly. Thicken endometrium may associate with intrauterine pathologies such as polyps, fibroids or hyperplasia may have an adverse effect on the implantation and clinical pregnancy (25). Therefore, in women with an abnormally thickened endometrium, it is necessary to carry out further investigations, such as a hysteroscopy to make clear and treat any intra-uterine lesions (6).

The observations in our study were based on fresh embryo transfer in IVF stimulated cycles, and our conclusions might not apply to frozen embryo transfer in HRT cycles. BU.Z et al. (26) retrospectively analyzed data from 2997 patients undergoing their first FET cycles, after adjusting for age, body mass index (BMI), baseline follicle stimulating hormone (FSH), FET protocol and No. of embryos transferred, the associations between EMT and clinical pregnancy rate (aOR: 1.39; 95% CI: 1.10-1.77, p < 0.01) and live birth rate (aOR: 1.50; 95% CI: 1.16-1.95, p < 0.01) were significant with thickness of 9-13mm. In another retrospective study of 1,627 FET cycles on patients with different EMT, Zhang, Q. et al., (17). found a non-linear relationship between EMT and pregnancy outcomes and an EMT threshold of 10.9 mm. The effect size of the left and right sides of the threshold were 1.16 (95% CI 1.07-1.25) and 0.89 (95% CI 0.78-1.01), respectively. In our previous study (10), we found in FET cycles, live birth rate would be optimal when the EMT was within the range of 8.7-14.5 mm. We hypothesized that the different threshold value between fresh cycle and FET cycle might be caused by confounders such as high levels of estrogen, large amount of Gn dosage or pre-increasing LH surge that would affect endometrial receptivity to some degree. Thus in FET cycles, the endometrium is more receptive and EMT threshold value range is wider than those in fresh cycles.

However, there are some limitations in this study. Firstly, this study was retrospective and there might be some confounders that we did not control for. In addition, we prioritized a few select pregnancy outcomes and did not investigate neonatal outcomes such as gestational age and birth weight. Finally, the observations in our study were based on fresh cycles in IVF/ICSI treatment, the conclusions of which might not be able to apply to FET cycles. In the future, a well-designed and randomized clinical trial would be needed for further study.

In conclusion, EMT has a curvilinear relationship with pregnancy outcomes in fresh embryo transfer cycles. Clinical pregnancy rate and live birth rate may achieve the optimal level when EMT ≥ 12 mm, but some adverse pregnancy outcomes would be observed when EMT ≥15 mm especially for clinical pregnancy. These would be relevant evidence when considering the embryo transfer.

## Data Availability Statement

The original contributions presented in the study are included in the article/**Supplementary Material**. Further inquiries can be directed to the corresponding authors.

## Ethics Statement

Written informed consent was obtained from the individual(s) for the publication of any potentially identifiable images or data included in this article.

## Author Contributions

CZ and XL supervised the entire study, including procedures, conception, design, and completion. SZ, LJ, YM, JS, and RH were responsible for collecting information. JX and SZ contributed to the analysis data and drafted the manuscript. XH participated in revising the article. JX and SZ contributed equally to this article. All authors contributed to the article and approved the submitted version.

## Funding

The National Natural Science Foundation of China (U2004130); The Major Projects in Provincial and National Union Construction of Henan Medical Science Research Plan (SBGJ202001002); The Provincial Natural Science Foundation of Henan (202300410456).

## Conflict of Interest

The authors declare that the research was conducted in the absence of any commercial or financial relationships that could be construed as a potential conflict of interest.

## Publisher’s Note

All claims expressed in this article are solely those of the authors and do not necessarily represent those of their affiliated organizations, or those of the publisher, the editors and the reviewers. Any product that may be evaluated in this article, or claim that may be made by its manufacturer, is not guaranteed or endorsed by the publisher.
