# Supplementary material for: The Effects of Endometrial Thickness on Pregnancy Outcomes of Fresh IVF/ICSI Embryo Transfer Cycles: An Analysis of Over 40,000 Cycles Among Five Reproductive Centers in China
Source: Front Endocrinol (Lausanne). 2022 Jan 24;12:788706. doi: 10.3389/fendo.2021.788706 (PMC8818785; doi:10.3389/fendo.2021.788706)
Supplement: Supplementary file 1 [file DataSheet_1.docx]

*Supplemental table 1.*

Characteristics and pregnancy outcomes between different COS protocol

| COS protocls | GnRH-agonist long-protocol | GnRH-antagonist protocol | Standardize diff. | P-value |
| --- | --- | --- | --- | --- |
| N | 34388 | 7744 |  |  |
| EMT on hcg  trigger day (mm) | 11.77 (2.45) | 10.89 (2.30) | 0.37 (0.35, 0.40) | <0.001 |
| E2 level on trigger  day(pg/ml) | 2464.00 (1495.00-3853.75) | 1492.00 (933.24-2404.00) | 0.66 (0.64, 0.69) | <0.001 |
| No. of retrieved  oocytes | 10.00 (7.00-13.00) | 7.00 (5.00-10.00) | 0.58 (0.56, 0.61) | <0.001 |
| Gn duration(day) | 10.62（2.20） | 9.19（1.63） | 0.74 (0.71, 0.76) | <0.001 |
| Clinical pregnancy | 20474 (59.54%) | 3574 (46.15%) | 0.27 (0.25, 0.30) | <0.001 |
| Live birth | 15938 (46.35%) | 2305 (29.76%) | 0.35 (0.32, 0.37) | <0.001 |
| Miscarriage | 2539 (12.40%) | 500 (13.99%) | 0.05 (0.01, 0.08) | 0.008 |

*Supplemental Figures*


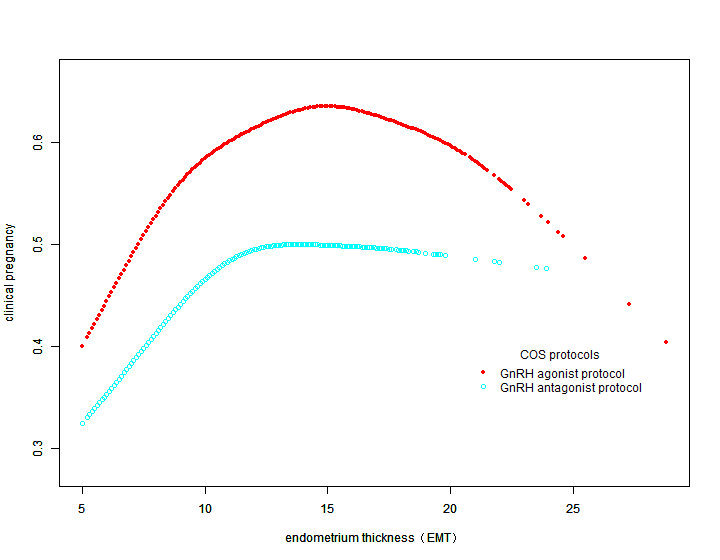


Supplemental Figure.1 Association between EMT and clinical pregnancy rate with different COS protocol.


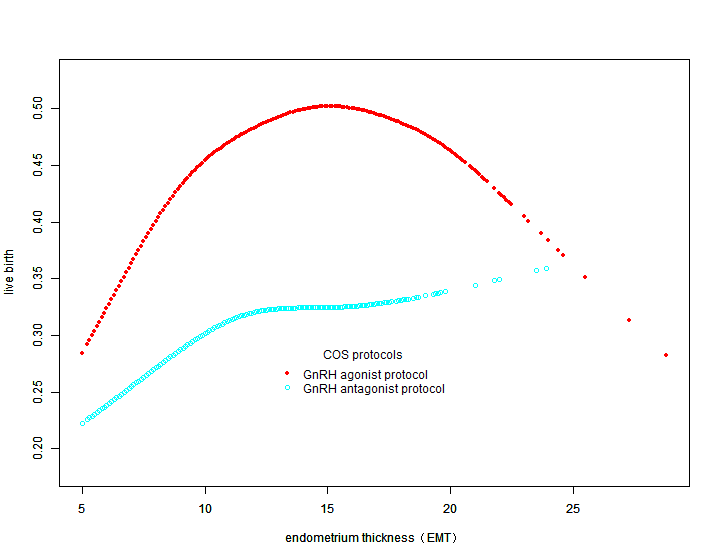


Supplemental Figure.2 Association between EMT and live birth rate with different COS protocol.
